# p19ARF is a critical mediator of both cellular senescence and an innate immune response associated with MYC inactivation in mouse model of acute leukemia

**DOI:** 10.18632/oncotarget.2969

**Published:** 2015-01-13

**Authors:** Alper Yetil, Benedict Anchang, Arvin M. Gouw, Stacey J. Adam, Tahera Zabuawala, Ramya Parameswaran, Jan van Riggelen, Sylvia Plevritis, Dean W. Felsher

**Affiliations:** ^1^ Division of Oncology, Departments of Medicine and Pathology, Molecular Imaging Program, Stanford University, Stanford, California, United States of America; ^2^ Department of Radiology, Stanford University, Stanford, California, United States of America

**Keywords:** MYC, p19ARF, ALL, senescence, macrophage

## Abstract

MYC-induced T-ALL exhibit oncogene addiction. Addiction to MYC is a consequence of both cell-autonomous mechanisms, such as proliferative arrest, cellular senescence, and apoptosis, as well as non-cell autonomous mechanisms, such as shutdown of angiogenesis, and recruitment of immune effectors. Here, we show, using transgenic mouse models of MYC-induced T-ALL, that the loss of either p19ARF or p53 abrogates the ability of MYC inactivation to induce sustained tumor regression. Loss of p53 or p19ARF, influenced the ability of MYC inactivation to elicit the shutdown of angiogenesis; however the loss of p19ARF, but not p53, impeded cellular senescence, as measured by SA-beta-galactosidase staining, increased expression of p16INK4A, and specific histone modifications. Moreover, comparative gene expression analysis suggested that a multitude of genes involved in the innate immune response were expressed in p19ARF wild-type, but not null, tumors upon MYC inactivation. Indeed, the loss of p19ARF, but not p53, impeded the *in situ* recruitment of macrophages to the tumor microenvironment. Finally, p19ARF null-associated gene signature prognosticated relapse-free survival in human patients with ALL. Therefore, p19ARF appears to be important to regulating cellular senescence and innate immune response that may contribute to the therapeutic response of ALL.

## INTRODUCTION

Acute T-cell lymphoblastic leukemia (T-ALL) is a common pediatric malignancy. In 20% of childhood ALL cases, chemotherapy and radiotherapy fail to result in sustained remission that in turn is associated with increased mortality [1]. Targeted inactivation of specific oncogenes may be a more specific and effective treatment for T-ALL.

The human c-MYC (MYC) oncogene is commonly overexpressed in human T-ALL [2]. We have made conditional transgenic mouse models of T-ALL [3] to demonstrate that inactivation of MYC induces sustained tumor regression, through the phenomenon termed “oncogene addiction” [4, 5]. In our model, MYC inactivation is associated with proliferative arrest, apoptosis, differentiation, and cellular senescence of the tumor, as well as with the shutdown of angiogenesis in the host [3, 6–8]. Thus, MYC-induced hematopoietic tumorigenesis is reversible.

However, in some cases, tumors reoccur through acquisition of additional genomic abnormalities [9]. Nonetheless, the eventual restoration of MYC overexpression appears to be required to fully regain a neoplastic phenotype [10]. Indeed, conditional inactivation of oncogenes in mouse models can be associated with tumor recurrence suggesting these models can be used to simulate therapeutic resistance [11, 12]. Previously, it has been identified that loss of specific tumor suppressors such as p53 (TRP53) can impede the ability of oncogene inactivation to induce sustained tumor regression [7, 8, 12, 13].

The genetic or epigenetic inactivation of INK4A (CDKN2A) locus encoding the tumor suppressors p16INK4A and p14ARF has been frequently implicated in the pathogenesis of human cancer [14, 15]. Notably, p14ARF has been shown to be lost in human lymphomas [16]. Similarly, the loss of p14ARF's murine homolog, p19ARF, has been shown to accelerate MYC-induced tumorigenesis associated with a decrease in apoptosis [12, 17]. Hence, ARF is thought to play a critical role in the tumorigenesis, largely through an influence on apoptosis. However, other reports suggest that p19ARF may regulate self-renewal and cellular senescence programs [18–21]. Less clear is whether p19ARF's influence on self-renewal or senescence contributes to MYC-induced tumorigenesis.

p19ARF is known to mediate many of its effects through p53-dependent mechanisms [15, 22]. However, a variety of p53-independent effects of p19ARF have been described [14, 23, 24]. In particular, p19ARF appears to directly bind to MYC, DDX5, and/or MIZ1 and alter their transactivation and transrepression functions/abilities [25–28]. In turn, p19ARF's function is influenced by the level of MYC expression [29]. Thus, p19ARF may influence MYC-induced tumorigenesis through multiple mechanisms.

Previously, we have examined the influence of loss of p53 expression on the ability of MYC inactivation to induce sustained tumor regression [8]. The loss of p53 expression prevented sustained tumor regression. Tumors that were p53+/− or −/− both reoccurred upon MYC inactivation, and all tumors exhibited loss of p53 protein expression. Surprisingly, we did not observe differences in proliferative arrest or apoptosis. Instead, tumors that were p53−/− exhibited a reduction in the shutdown of angiogenesis upon MYC inactivation. Thus, the loss of p53 impedes the ability of MYC inactivation to switch off angiogenesis.

Here we examined the relative influence of p19ARF or p53 loss on the ability of MYC inactivation to elicit sustained tumor regression in a conditional transgenic mouse model of T-ALL. The loss of p19ARF or p53 completely impeded the ability of MYC inactivation to induce sustained tumor regression. Neither gene influenced the proliferation or apoptosis upon MYC inactivation. The loss of p53, and less so p19ARF, were associated with a change in the ability to suppress angiogenesis. In contrast, the loss of p19ARF, but not p53, greatly impeded the ability to induce cellular senescence. Moreover, p19ARF, but not p53, prevented the recruitment of macrophages to the tumor site. Finally, comparative gene expression analysis of MYC-induced tumors with or without loss of p19ARF revealed a gene signature associated with genes that are known to recruit innate immune effectors and that could also prognosticate relapse-free survival in human patients with ALL. Our work suggests a role of p19ARF in the mechanism by which MYC inactivation elicits an innate immune response and cellular senescence that may be important to sustained tumor regression.

## RESULTS

### Effects of p19ARF or p53 loss in initiation and maintenance of MYC-induced tumors

**Figure 1:**
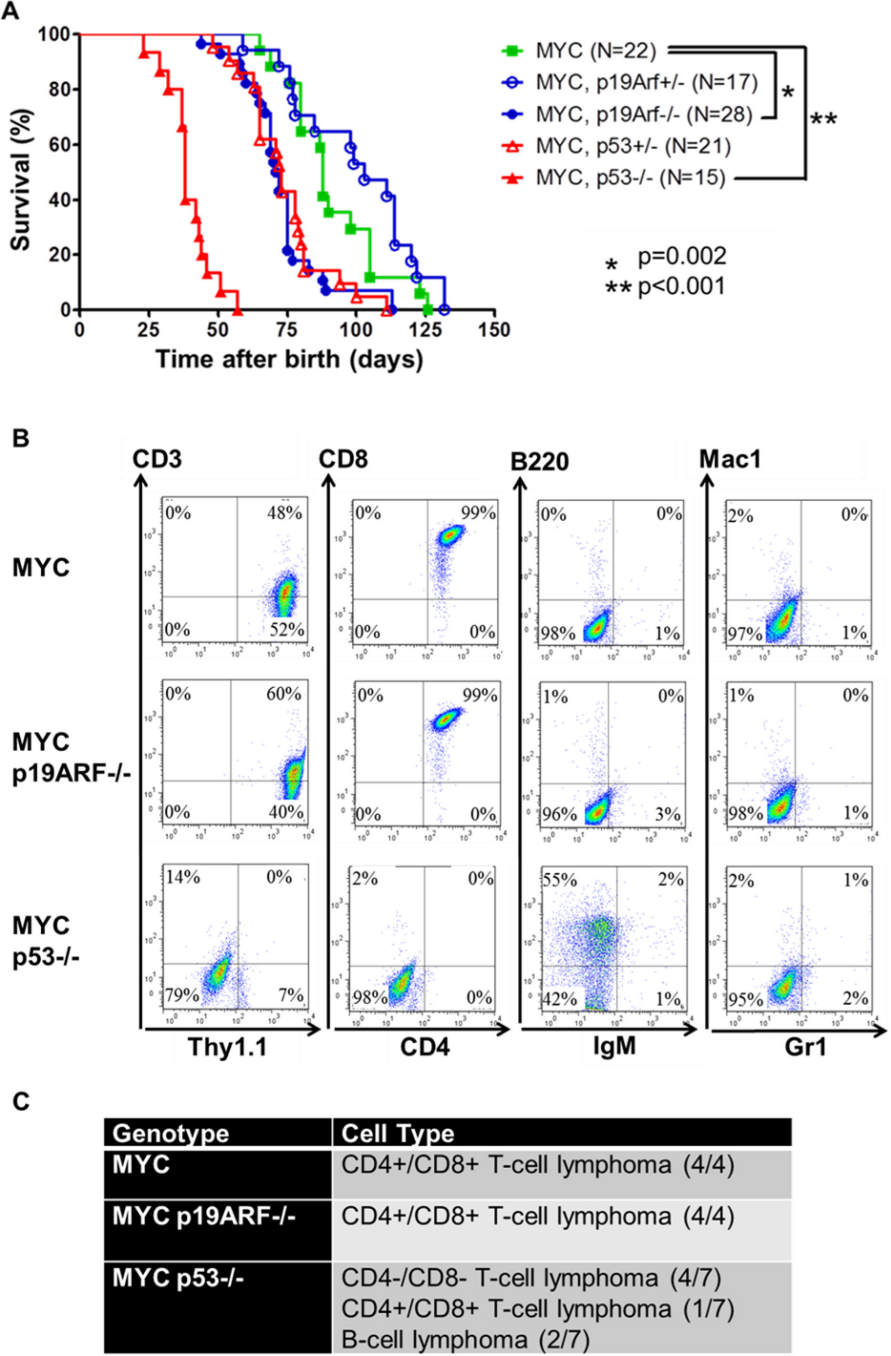
Loss of p19ARF or p53 cooperates with MYC **(A)** Survival plot showing that loss of both alleles of p19ARF or loss of one or two alleles of p53 accelerates lymphomagenesis. MYC mice develop lymphoma in 89 days (*n* = 22), while MYC p19ARF+/− mice in 100 days (*n* = 17, *p* = 0.1), MYC p19ARF−/− mice in 73 days (*n* = 28, *p* = 0.002), MYC p53−/− mice in 40 days (*n* = 15, *p* = 0.001), MYC p53+/− mice in 74 days (*n* = 21, 0.004). **(B)** Flow cytometry analysis of cell surface markers of lymphomas from MYC, MYC p19ARF−/−, MYC p53−/−. The cells were analyzed by FACS for Thy1.1, CD2, CD4, CD8, B220, IgM, Gr1 and Mac1. **(C)** Lymphomas in MYC (*n* = 4) and MYC p19ARF−/− mice (*n* = 4) were CD4+/CD8+ T-cell lymphoma, while those in MYC p53−/− mice (*n* = 7) were CD4+/CD8+ T-cell lymphoma (14%), CD4−/CD8− T-cell lymphoma (56%) or B-cell lymphoma (28%).

In humans, MYC oncogene is frequently overexpressed in Burkitt's, T-cell, and B-cell lymphomas. Loss of ARF or p53 expression in lymphomas has been associated with poor prognosis. Here, using transgenic mice, we examined the influence of the loss of p19ARF or p53 on the ability of MYC inactivation to induce sustained tumor regression. Previously, we had described the use of the tetracycline regulatory system to conditionally express human c-MYC transgene under the control of Eμ heavy chain enhancer and SR-α promoter (Eμ-tTA TRE-MYC) [3]. We generated p19ARF−/− Eμ-tTA TRE-MYC mice (referred to as MYC p19ARF−/− mice) and p53−/− Eμ-tTA TRE-MYC mice (referred to as MYC p53−/− mice) to study the role of tumor suppressors p19ARF and p53 in MYC-induced lymphoma. MYC p19ARF−/− and MYC p53−/− mice, compared to MYC (Eμ-tTA TRE-MYC) transgenic mice, exhibited shorter latencies of tumor onset (73 versus 89 days, *p* = 0.002; 40 versus 89 days, *p* = 0.001) (Figure 1A). Hence, the loss of p53 or p19ARF cooperates with MYC overexpression to induce T-ALL. Note, we confirmed that in tumor from mice knocked-out for either p19ARF or p53, there was continued expression of the wild-type tumor suppressor (Supplementary Figure 1A). Our results are similar to those described in the Eμ-MYC model of B-cell lymphoma [17, 30].

The tumors were characterized by flow cytometry for surface markers of hematopoietic lineages (Figure 1B). Our MYC mice historically all develop CD4+/CD8+ T-cell lymphoma. In the MYC p19ARF−/− mice, four tumors analyzed were also CD4+/CD8+ T-cell lymphoma (Figure 1C). In MYC p53−/− mice, only one out of seven tumors were CD4+/CD8+ T-cell lymphoma, four were CD4-/CD8-, and the remaining two were weakly CD3+ and B220+, but CD4-/CD8-/IgM-. Hence, the loss of p53 may allow MYC to transform more immature hematopoietic cells compared to loss of p19ARF.

**Figure 2:**
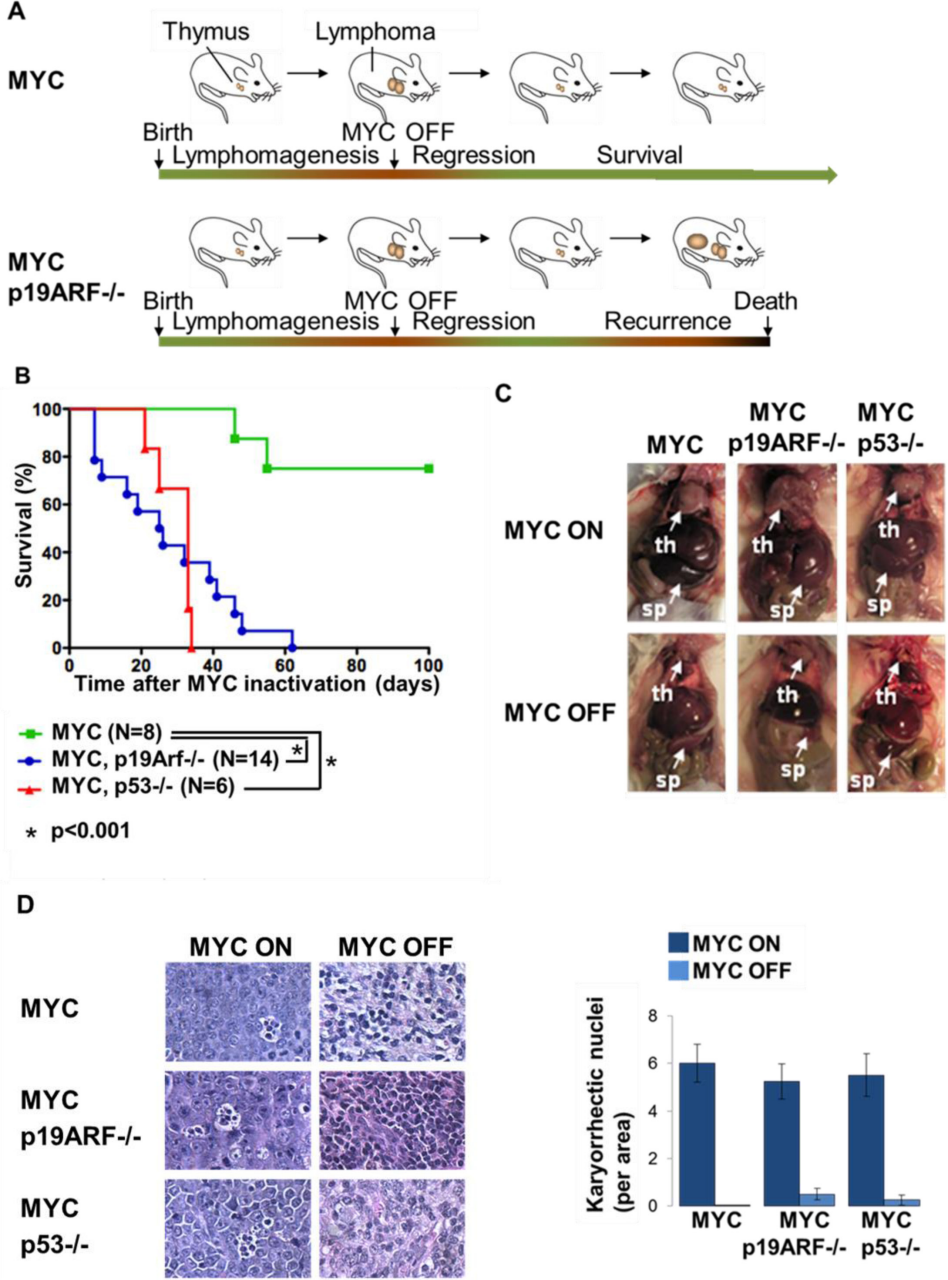
Loss of p19ARF or p53 facilitates lymphoma recurrence **(A)** Schematic showing the experimental design. MYC and MYC p19ARF−/− mice develop lymphomas when MYC is expressed from the time of conception. MYC is turned off when the mice develop lymphoma. The mice are monitored for lymphoma recurrence following the initial lymphoma regression. **(B)** Survival curve showing the percentage of mice versus time from MYC inactivation to death. Only 25% of MYC mice (*n* = 8) had spontaneous recurrence of lymphoma, while 100% of the MYC p19ARF+/− (*n* = 14), MYC p19ARF−/− (*n* = 14), MYC p53+/− (*n* = 14) and MYC p53−/− (*n* = 6) mice had recurrence. The survival time after MYC inactivation was 27 days in MYC p19ARF−/− mice, which was shorter than the survival time in MYC p19ARF+/− mice (35 days, *p* = 0.094) and MYC p53+/− mice (49 days, *p* = 0.014), but was not different from the survival time in MYC p53−/− mice (30 days, *p* = 0.6). **(C)** MYC, MYC p19ARF−/− and MYC p53−/− mice show similar pathology with enlarged thymus and spleen due to lymphoma. Upon 5 days of MYC inactivation, thymus and spleen shrink. **(D)** Hematoxylin-eosin staining shows that lymphoma in the thymuses of MYC, MYC p19ARF−/− and MYC p53−/− mice show similar histology at the microscopic level. The lymphoma sections include many apoptotic cells with fragmented nuclei. Upon 5 days of MYC inactivation, we note decrease in apoptotic cells.

Next, we examined the influence of loss of p19ARF or p53 on tumor recurrence after MYC inactivation (Figure 2A). Upon MYC inactivation, all tumors initially regressed as apparent by reduced abdominal girth and reduced palpable lymphadenopathy. Subsequently, MYC tumors recurred at a rate of 25% within 100 days of observation, while p19ARF−/− and p53−/− tumors reoccurred at 100% (Figure 2B). Notably, MYC, MYC p19ARF−/−, or MYC p53−/− tumors exhibited similar macroscopic pathology with enlarged thymus, spleen, and lymph nodes (Figure 2C), as well as microscopic pathology with similar numerous karyorrhectic nuclei that were reduced upon MYC suppression (Figure 2D). Thus, even though MYC p19ARF−/− and MYC p53−/− tumors looked similar to MYC tumors, all of these tumors recurred.

### Influence of loss of p53 or p19ARF on MYC-inactivation *in vitro*

**Figure 3:**
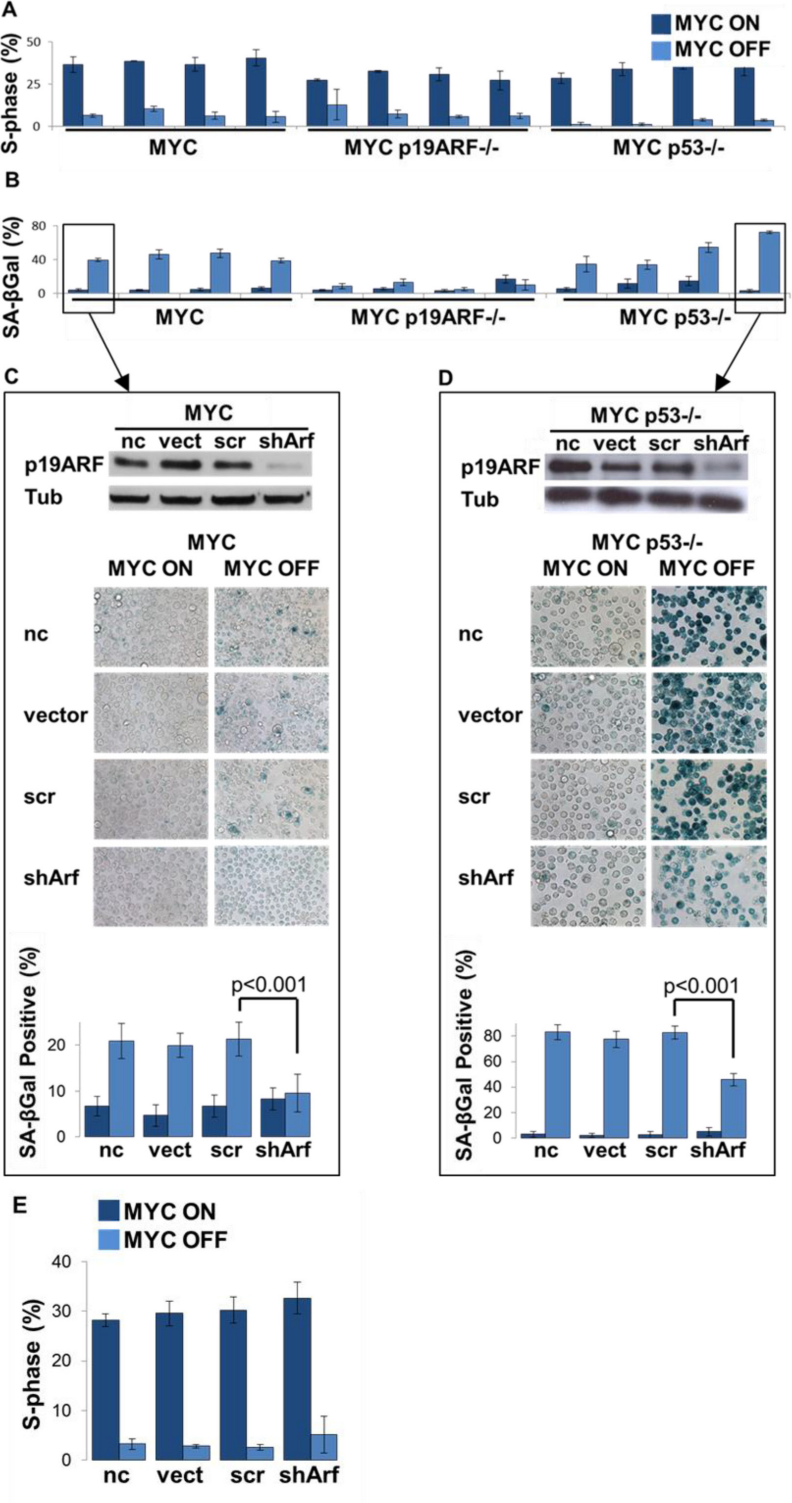
Loss of p19ARF prevents MYC inactivation induced senescence in a p53-independent manner **(A)** MYC inactivation induces proliferative arrest in MYC, MYC p19ARF−/− and MYC p53−/− lymphoma cells. Graph shows that after 2 days of MYC inactivation *in vitro*, S-phase fraction measured by flow cytometry on PI-stained cells drops to 7%, 8% and 3% in MYC, MYC p19ARF−/− and MYC p53−/− cells, respectively. **(B)** MYC inactivation induces increased SA-beta-galactosidase activity in MYC and MYC p53−/− lymphoma cells, but not in MYC p19ARF−/− cells. Graph shows that upon MYC inactivation percentage of cells that are SA-beta-galactosidase positive increased in MYC MYC p53−/− cells. SA-beta-galactosidase positivity was much lower in MYC p19ARF−/− cells and was unchanged before or after MYC inactivation. **(C)** Western blot showing knockdown of p19ARF expression by shRNA in a MYC p53−/− lymphoma cell line. Alpha-tubulin serves as loading control. shRNA targets exon1β of CDKN2A, hence it is specific to p19ARF. **(D)** Knockdown of p19ARF even in the absence of p53 curbs the increase in SA-beta-galactosidase activity when MYC is turned off. One MYC p53−/− cell line was transfected with an empty vector, a vector expressing control shRNA or p19ARF shRNA. Cells were stained with SA-beta-galactosidase assay in MYC on condition or 2 days after MYC inactivation. Graph showing percentage of cells that are positive for SA-beta-galactosidase activity. After 2 days of MYC inactivation, p19ARF knockdown cells (shArf) had significantly lower percentage of SA-beta-galactosidase positive cells as compared to uninfected (*p* = 0.001), empty vector (*p* = 0.001) and control shRNA cells (*p* = 0.001). **(E)** PI staining of S-phase cell cycle data, revealing that MYC p53−/− tumors do not demonstrate cell cycle arrest upon knockdown of p19ARF with shRNA.

MYC inactivation in tumors elicits both *in vitro* and *in vivo* proliferative arrest, apoptosis, and senescence [3, 7, 8]. MYC inactivation *in vitro* was associated with comparable proliferative arrest (G1 arrest) in MYC, MYC p19ARF−/−, and MYC p53−/− cells as measured by FACS analysis of PI stained cells (S-phase: 7%, 8% and 3%, respectively) (Figure 3A).

Second, MYC and MYC p53−/−, but not MYC p19ARF−/− cells exhibited senescence upon MYC inactivation, as assayed by SA-beta-galactosidase activity. SA-beta-galactosidase activity increased from 5% to 43% in MYC and from 9% to 49% in MYC p53−/−, while it remained unchanged at 8% and 9% in MYC p19ARF−/− before and after MYC inactivation (Figure 3B; MYC OFF SA-beta-galactosidase activity, MYC versus MYC p19ARF−/−, *p* < 0.001; MYC versus MYC p53−/−, *p* = 0.5; MYC p19ARF−/− versus MYC p53−/−, *p* = 0.021). Acute knockdown of p19ARF expression by shRNA resulted in decreased SA-beta-galactosidase activity (Figure 3C). Thus, regardless of the status of p19ARF or p53, MYC inactivation induces proliferative arrest, but differential effects on cellular senescence.

p19ARF has been shown to mediate its effects through p53-dependent mechanisms [15, 22]. To determine if loss of p19ARF abrogated MYC inactivation-induced senescence independent of p53, p19ARF expression was suppressed with an shRNA in a MYC p53−/− cell line (Figure 3D, top). Suppression of p19ARF, even in the absence of p53, impeded cellular senescence (83% versus 46% in control shRNA and p19ARF knockdown cells, respectively, *p* < 0.001) (Figure 3D). However, concomitant deficiency of p19ARF and p53 did not rescue the ability of MYC inactivation to induce cell cycle arrest (Figure 3E, S phase was 5.2% in p19ARF/p53 deficient cells after MYC inactivation). Therefore, loss of p19ARF appears to abrogate cellular senescence upon MYC inactivation through a mechanism that is independent of p53.

### Influence of loss of p53 or p19ARF on MYC-inactivation *in vivo*

**Figure 4:**
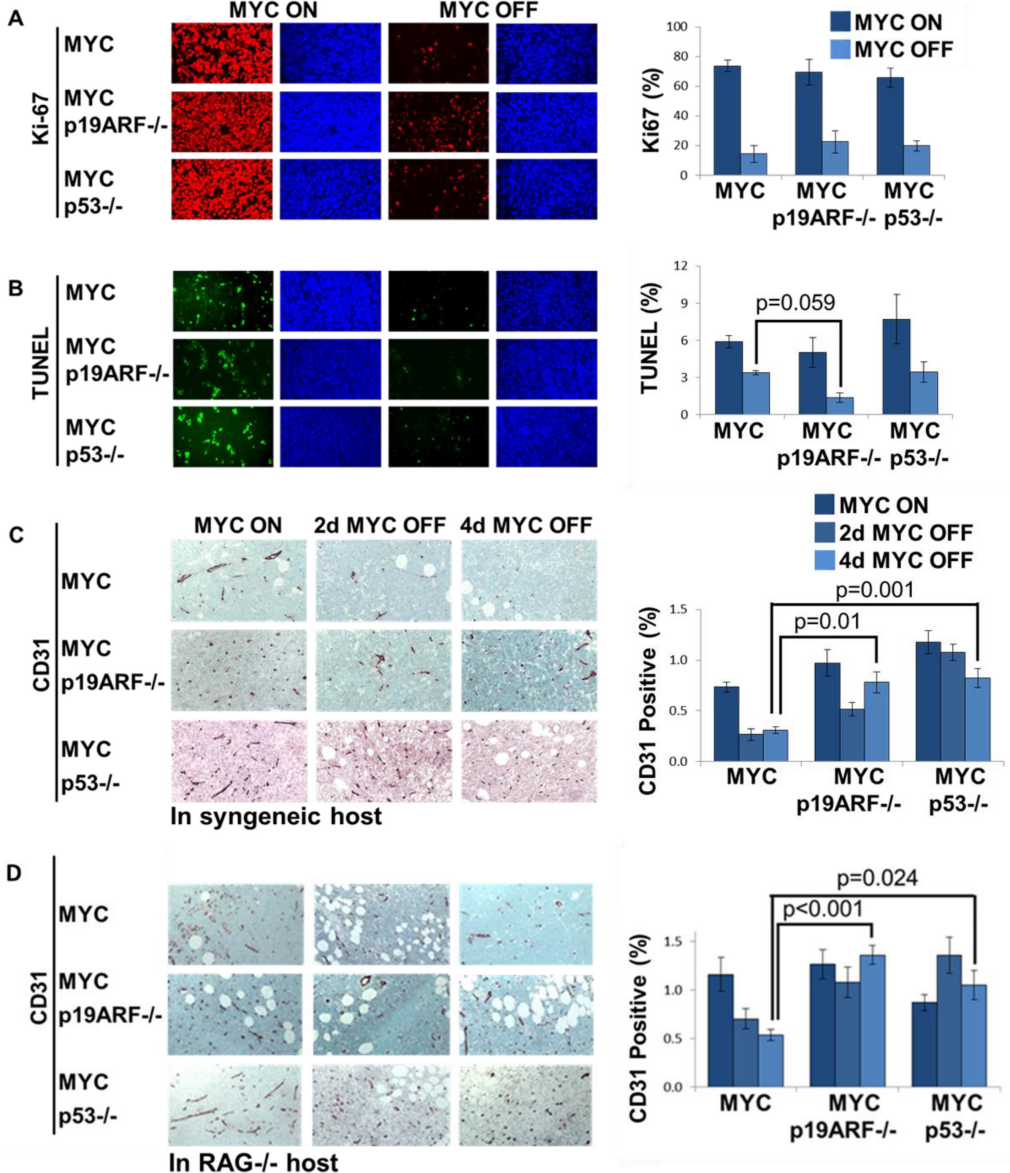
Shutdown of angiogenesis, but not proliferative arrest, upon MYC inactivation *in vivo* is diminished by the loss of p19ARF or p53 **(A)** Proliferation analysis by Ki67 immunofluorescence on sections of primary lymphoma. Graph shows Ki67 positive area as normalized to DAPI. Upon 5 days of MYC inactivation, Ki67 positive cells decreased from 74%, 69%, 66% to 14%, 22%, 20% in MYC, MYC p19ARF−/− and MYC p53−/− lymphoma, respectively. There was no significant difference between MYC p19ARF−/− and MYC (MYC off, *p* = 0.4) or MYC p53−/− and MYC lymphomas (MYC off, *p* = 0.5). MYC inactivation leads to a decrease in proliferation in MYC, MYC p19ARF−/− and MYC p53−/− lymphomas. **(B)** Apoptosis analysis by TUNEL staining on sections of primary lymphoma. MYC inactivation leads to a decrease in apoptosis in MYC, MYC p19ARF−/− and MYC p53−/− lymphomas. Graph shows TUNEL positive area as normalized to DAPI. Upon 5 days of MYC inactivation, TUNEL positive cells decreased in MYC, MYC p19ARF−/− and MYC p53−/− lymphoma. There was no significant difference between MYC p53−/− and MYC lymphomas (MYC off, *p* = 0.9) or between MYC p19ARF−/− and MYC lymphomas (MYC off, *p* = 0.059). **(C)** Tumor vessel density assessed by CD31 immunohistochemistry on transplanted lymphoma cell lines in syngeneic hosts before and during MYC inactivation time course. Graph shows percentage of CD31 positively stained area. MYC tumors exhibit dramatically reduced CD31 staining upon MYC inactivation. MYC p19ARF−/− and MYC p53−/− tumors show elevated CD31 staining compared to MYC tumors both before and after MYC inactivation. **(D)** Tumor vessel density assessed by CD31 immunohistochemistry on transplanted lymphoma cell lines in immunodeficient RAG−/− hosts.

We examined the influence of the loss of p53 or p19ARF on MYC inactivation *in vivo* in an immune intact host. Similar to our *in vitro* results, MYC inactivation *in vivo* was associated with no significant difference proliferative arrest observed in MYC, MYC p19ARF−/−, and MYC p53−/− tumors measured by the percentage of Ki67 positively staining cells in MYC inactivated tumors (14%, 22%, and 20%, respectively (MYC versus MYC p19ARF−/−, not significant (n.s.); MYC versus MYC p53−/−, n.s) (Figure 4A). MYC inactivation was also associated with no significant differences in apoptosis in MYC, MYC p19ARF−/− or MYC p53−/− tumors as measured by the TUNEL assay at 5 days post MYC inactivation (1.4% in MYC p19ARF−/− versus 3.3% in MYC, *p* = 0.059, 3.4% in MYC p53−/− versus 3.3% in MYC, n.s.) (Figure 4B). Thus, loss of p53 or p19ARF did not influence proliferative arrest or apoptosis observed upon MYC inactivation in lymphomas *in vivo*.

MYC inactivation also results in the shutdown of angiogenesis as measured by a decrease in mean vessel density that is impeded in p53−/− tumors [8]. Similarly, transplanted MYC p19ARF−/− tumors exhibited a blunted reduction in mean vessel density upon MYC inactivation (Figure 4C) (MYC p53−/− versus MYC tumors, *p* < 0.001; MYC p19ARF−/− versus MYC tumors *p* = 0.010). Therefore, the loss of either p53 or p19ARF attenuates the shutdown of angiogenesis upon MYC inactivation.

**Figure 5:**
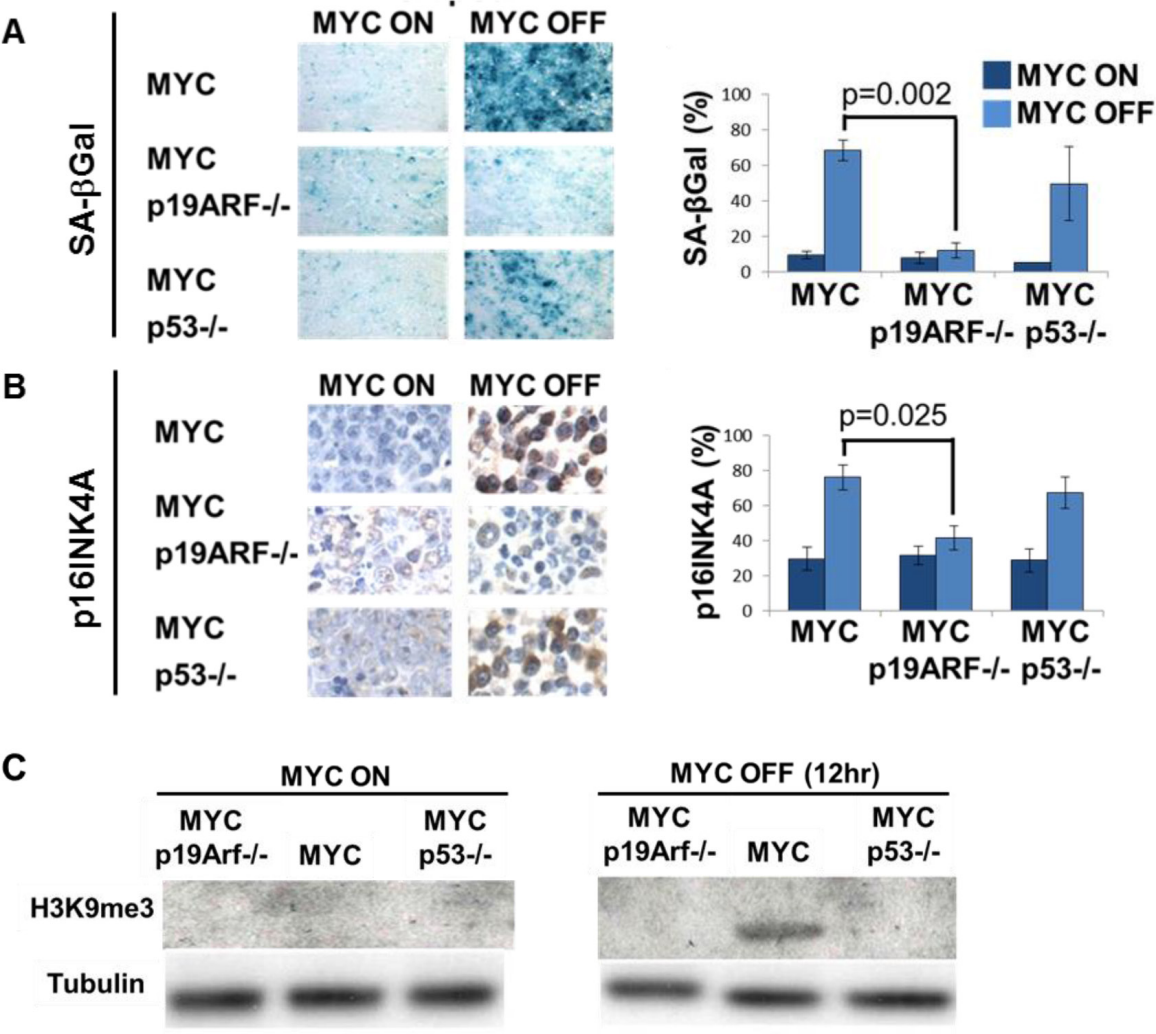
Loss of p19ARF prevents MYC inactivation induced senescence *in vivo* **(A)** SA-beta-galactosidase staining on sections of primary lymphoma. MYC inactivation induces SA-beta-galactosidase activity in MYC and MYC p53−/−, but not MYC p19ARF−/− lymphoma. Graph shows quantitation of SA-beta-galactosidase positivity. After MYC inactivation, MYC p19ARF−/− lymphoma had significantly lower SA-beta-galactosidase activity than MYC lymphoma (*p* = 0.002), while MYC p53−/− lymphoma was not significantly different from MYC lymphoma (*p* = 0.3). **(B)** p16INK4A immunohistochemistry on sections of primary lymphoma. MYC inactivation induces an increase in p16INK4A levels in MYC lymphoma, and to a lesser extent, in MYC p53−/− lymphoma, but not in MYC p19ARF−/− lymphoma. Graph shows quantitation of p16INK4A expression. After MYC inactivation, MYC p19ARF−/− lymphoma had significantly lower p16INK4A expression than MYC lymphoma (*p* = 0.025), while MYC p53−/− lymphoma was not significantly different from MYC lymphoma (*p* = 0.4). **(C)** H3K9 trimethylation increased upon MYC inactivation in MYC tumors, but not in MYC p53−/− and MYC p19ARF−/− tumors as measured by Western blot.

Cellular senescence also occurs upon MYC inactivation and contributes to tumor regression [31]. Following MYC inactivation, SA-beta-galactosidase positivity in MYC p19ARF−/− tumor sections was much lower than in MYC or MYC p53−/− tumor sections (12% in MYC p19ARF−/−versus 68% in MYC, *p* = 0.002, 49% in MYC p53−/− versus 68% in MYC, n.s.) (Figure 5A). The p16INK4A immunohistochemical staining in p19ARF−/−, but not in MYC p53−/−, tumor sections was significantly reduced than in MYC tumor sections (41% in MYC p19ARF−/− versus 76% in MYC, *p* = 0.025; 67% in MYC p53−/− versus 76% in MYC, n.s.) (Figure 5B). Notably, the induction of p16INK4A upon MYC inactivation appears to occur through an increase in protein but not mRNA (Supplementary Figure 1B). Upon MYC inactivation, H3K9 trimethylation increased in MYC tumors, but not in MYC p53−/− or MYC p19ARF−/− tumors, suggesting the impairment of cellular senescence (Figure 5C). Thus, p19ARF, and to a less extent p53, are required to induce cellular senescence upon MYC inactivation.

The host immune system is required for MYC inactivation to elicit both cellular senescence and the shutdown of angiogenesis [32]. Notably, the loss of p19ARF or p53 further blunted the shutdown of angiogenesis, as is the case in transplanted tumors in syngeneic hosts. In immunodeficient hosts, the shutdown of angiogenesis was less pronounced in transplanted MYC tumors and was completely blocked in MYC p19ARF−/− and MYC p53−/− tumors (Figure 4D). Hence, p19ARF and p53 and host immune factors contribute to the mechanism of shutdown of angiogenesis upon MYC inactivation.

### p19ARF or p53 status has no major effect on the expression of MYC transactivation targets

**Figure 6:**
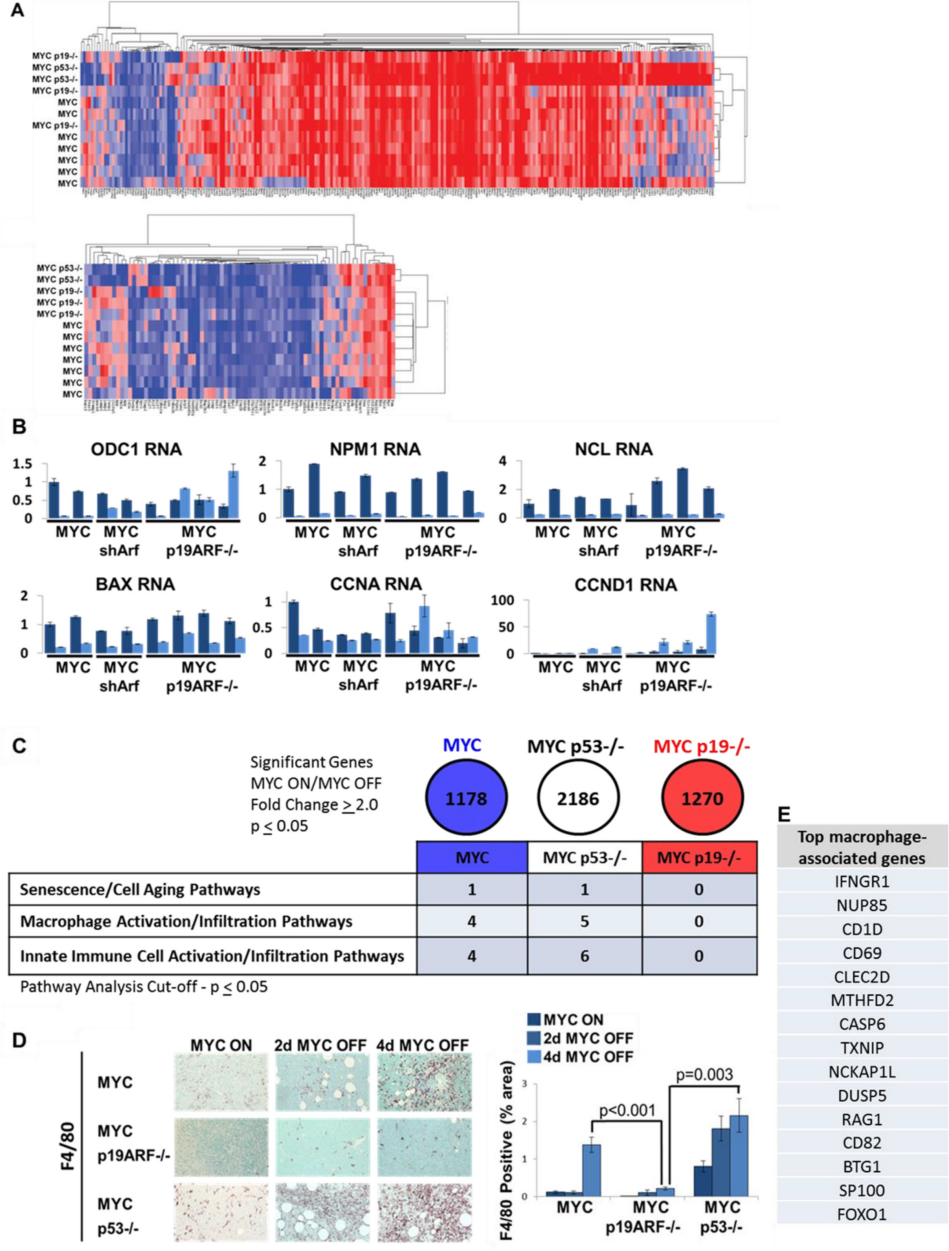
Microarray suggests deregulation of senescence and innate immune cell infiltration into MYC p19ARF−/− tumors Microarray analysis was performed before and after 24 hours of MYC inactivation in MYC, MYC p19ARF−/−, and MYC 53−/− lymphoma cells derived from primary tumors. **(A)** Hierarchal clustering of MYC transactivation targets (top panel) and hierarchal clustering of MYC transrepression targets (bottom panel). No global differences were seen in regulation of MYC target genes between MYC, MYC p19ARF−/−, and MYC p53−/−. **(B)** RT-qPCR results of specific MYC target genes. No significant difference was seen in the expression of these target genes between MYC, MYC p53−/−, and MYC p19ARF−/− tumors. **(C)** Summary table of key Genespring pathway analysis conducted on microarray results for significant differentially expressed genes in the MYC, MYC p53−/−, and MYC p19ARF−/− tumors. Senescence/cell aging pathway and innate immune cell activation/infiltration pathways were found to be significantly enriched in MYC and MYC p53−/− tumors, but not in MYC p19ARF−/− tumors. **(D)** Immunohistochemistry for F4/80, a marker for macrophages, performed on time course sections from transplanted tumors before and after MYC inactivation. MYC p19ARF−/− tumors exhibit dramatically reduced macrophage infiltration before and after MYC inactivation compared to MYC and MYC p53−/− tumors (MYC off *p* = 0.001 and *p* = 0.003, respectively). **(E)** List of genes associated with macrophages in MYC and MYC p53−/−, but not in MYC p19ARF−/−, tumors.

Previously, it has been described that ARF alters MYC transcriptional transactivation function [25, 33, 34], but not necessarily its transrepression [25]. p19ARF also appears to bind to MYC antagonist MIZ1, enhancing MYC transrepression [26]. The loss of p19ARF could influence senescence by modulating MYC's regulation of transcriptional transactivation and/or transrepression. We analyzed changes in gene expression by microarray upon 24 hours of MYC inactivation. We did not observe any global deregulation of either MYC transactivation or transrepression target genes in MYC p19ARF−/− lymphoma (Figure 6A). Using GSEA algorithm to more quantitatively compare MYC transactivation and transrepression targets to gene expression patterns in MYC, MYC p19ARF−/−, and MYC p53−/− tumors, we observed that MYC transrepression targets show modest differences in enrichment in MYC p19ARF−/− cells that did not achieve statistical significance. The absence of p19ARF or p53 leads to differential changes in the global transcriptome upon MYC inactivation; however, this effect is not specific to MYC targets (Supplementary Figure 2A and 2B). There is strong evidence that most of the MYC transactivation targets in the MYC tumors (88.9%) are also transactivated in the p19ARF−/− or MYC p53−/− tumors. (Supplementary Figure 2C). This quantitatively suggests that most of the transactivated targets in the MYC tumors remain upregulated in the p19ARF−/− or MYC p53−/− tumors. Overall, our results do not provide evidence that p19ARF or p53 specifically alters expression levels of MYC transactivation or transrepression targets.

Furthermore, by quantitative PCR, we examined for specific differences in the regulation of MYC transactivation target genes. Some MYC transactivation targets (ODC1 and CCNA) were very strongly downregulated only in MYC lymphoma cells, but not in MYC p19ARF−/− or MYC p19ARF shRNA knockdown cells (Figure 6B). However, other MYC transactivation targets (NPM, NCL1, and BAX) were downregulated upon MYC inactivation irrespective of p19ARF status. Upon MYC inactivation, transrepression target CCND1 remains repressed in MYC cells, but not in MYC p19ARF−/− cells. Thus, the presence of either MYC or p19ARF is sufficient to suppress the expression of CCND1. Thus, p19ARF loss induced only modest changes in transactivation by MYC.

### p19ARF status influences expression of senescence and innate immunity genes and infiltration of tumors by macrophages

To more generally examine the influence of p19ARF and p53 on gene expression, pathway analysis of the microarray data was performed for significantly expressed genes (SEG). Notably, in the MYC or MYC p53−/−, but not in MYC p19ARF−/− lymphomas, differences were found in a senescence/cell aging pathway, macrophage activation/infiltration and innate immune cell activation/infiltration pathways (Figure 6C). Indeed, immunostaining of macrophages for F4/80 revealed that upon MYC inactivation in transplanted lymphomas that were MYC or MYC p53−/− there was a significant increase in macrophage infiltration, this was not seen in p19ARF−/− tumors (Figure 6D). p19ARF loss in tumor cells, not in the host, is responsible for the decrease in macrophage infiltration. Hence, p19ARF in tumors appears to regulate the ability of MYC inactivation to recruit innate immune effectors. Similarly, our analysis of the microarray data identified differential changes in genes associated with the activation of macrophages (Figure 6E). Thus, p19ARF influenced the expression of many genes associated with the recruitment of an innate immune response.

Macrophage infiltration has been previously associated with cellular senescence of tumors [35]. p19ARF has been suggested to influence macrophage activation [36]. Hence, MYC p19ARF−/− lymphomas exhibit differences in gene expression programs associated with senescence and innate immunity, in turn associated with a difference *in vivo* in the recruitment of innate immune effectors.

### A gene signature predicts clinical outcome in human all patients

**Figure 7:**
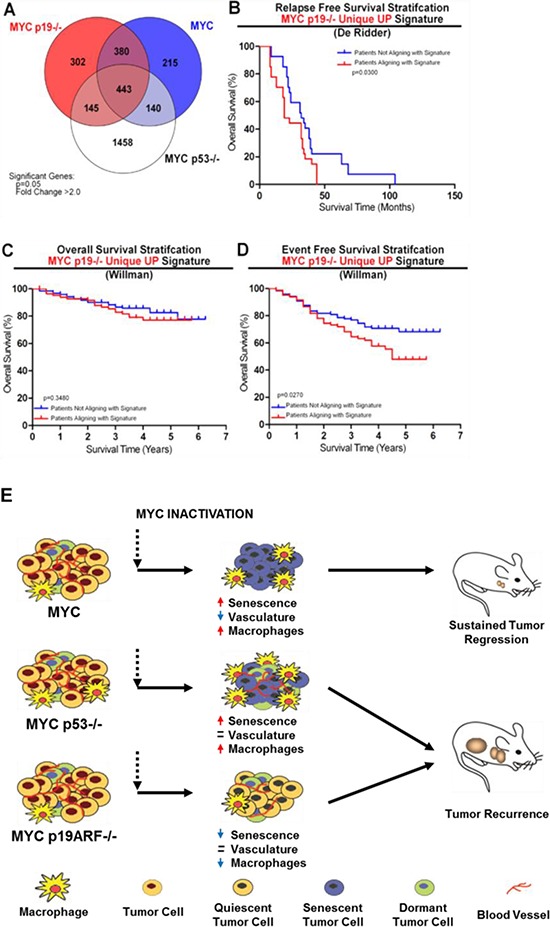
Microarray signature associated with loss of p19ARF predicts poor event free survival in B- and T-ALL patients and model showing the mode of recurrence of MYC p19ARF−/− and MYC p53−/− lymphomas **(A)** Venn diagram shows common and unique genes that are upregulated by MYC in MYC, MYC p19ARF−/− and MYC p53−/− mouse lymphomas. Genes that have a MYC on/MYC off expression ratio of +/− 2-fold in MYC, MYC p19ARF−/− and MYC p53−/− mouse lymphomas are shown. MYC p19ARF−/− unique gene signature was identified. **(B)** Human B- and T-ALL patients from the cohort collected by the de Ridder laboratory (GSE18497) for the Diagnosis of ALL Relapse study were divided into two groups by k-means clustering according to their expression of the genes within the MYC p19−/− unique gene signature. The two groups were then stratified via Kaplan-Meier survival analysis for relapse free survival, revealing that patients overexpressing the MYC p19−/− unique gene signature display a significantly lower relapse free survival than those that showed lower expression of the signature genes. **(C)** Human B- and T-ALL patients from the cohort collected by the Willman laboratory (GSE11877) for the Children's Oncology Group Study 9906 for High-Risk Pediatric ALL were divided into two groups by k-means clustering similar to the first data set and then stratified via Kaplan-Meier survival analysis for overall survival, revealing that there was no significant difference between the two patient groups based on overall survival. **(D)** The two groups were then stratified via Kaplan-Meier survival analysis for event free survival based on the same k-means clustering for C. This analysis revealed that patients overexpressing the MYC p19−/− unique gene signature showed a significantly lower event free survival (shorter time to relapse) than those that showed lower expression of the signature genes. **(E)** Model showing MYC, MYC p19ARF−/− and MYC p53−/− lymphomas respond to MYC inactivation differently. MYC lymphomas undergo sustained regression and remain in remission due to senescence of lymphoma cells and decrease in microvessel density. MYC p19ARF−/− and MYC p53−/− lymphomas both fail to undergo sustained regression and eventually reoccur; albeit through different mechanisms. MYC p19ARF−/− lymphomas reoccur through evasion of MYC inactivation induced senescence, while MYC p53−/− lymphomas reoccur through sustained angiogenesis despite MYC inactivation.

Loss of both p16INK4A and p14ARF are common events in human ALL, [16]. Although in humans p14ARF status has not yet been reported to prognosticate in ALL patients, it has been has been associated with poor prognosis in AML, squamous cell carcinoma, and osteosarcoma [37–39]. From the microarray analysis of MYC, MYC p19ARF−/−, and MYC p53−/− murine lymphomas, we identified a gene signature for each genotype (Figure 7A). When T- and B-ALL samples from a single patient cohort (GSE18497, 41 patients) were stratified using the MYC p19ARF−/− gene signature, patients who expressed high levels of the genes within this gene signature demonstrated significantly worse relapse-free survival (RFS, *p* = 0.031) (Figure 7B). Our signature was validated using a second dataset (GSE11877, 207 patients) demonstrating significantly reduced event-free survival (EFS, *p* = 0.027) (Figure 7D). However, this gene signature did not stratify patients significantly based on overall survival (Figure 7C). In contrast to MYC p19ARF−/− signature, MYC or MYC p53−/− gene signature was not prognostic for patient survival in these data cohorts (Supplementary Figure 3 A–D). We conclude that our gene signature can prognosticate relapse free survival in human ALL patients.

## DISCUSSION

We have found that the loss of p19ARF or p53 have distinct mechanistic consequences impeding the ability of MYC inactivation to induce sustained tumor regression. Notably, p19ARF loss, but not p53, loss was associated with the loss of cellular senescence and the inability to elicit an innate immune response upon MYC inactivation. We utilized comparative gene expression analysis to identify that p19ARF loss has broad influence on gene expression changes upon MYC inactivation. This signature included a multitude of genes associated with senescence and innate immune activation, and was found to be prognostic in human ALL. Our results may have implications for the general therapeutic response of human patients with T-ALL.

The loss of either p19ARF or p53 similarly cooperated with MYC overexpression to induce T-ALL, similar to what has been reported previously [8, 17, 25]. The resulting p19ARF or p53 negative tumors inevitably reoccurred upon MYC inactivation. In the absence of MYC, neither loss of p19ARF, nor p53, had any significant effect on proliferative arrest or apoptosis. The loss of p53, and to a lesser extent p19ARF, impeded the shutdown of angiogenesis upon MYC inactivation. However, strikingly, loss of p19ARF, but not p53, markedly impeded cellular senescence upon MYC inactivation, as measured by SA-beta-galactosidase, p16INK4A expression, and senescence-associated histone modifications. The loss of p19ARF but not p53 was also associated with a marked change in the expression of genes associated with innate immunity upon MYC inactivation. In turn, this correlated with the reduction in the recruitment of macrophages observed *in situ* upon MYC inactivation. Finally, a p19ARF-associated gene signature was prognostic for human patients with ALL (Figure 7E). Hence, our results suggest a difference in the mechanism by which p19ARF and p53 influence tumor recurrence upon oncogene inactivation.

Our observations are consistent with the notion that cellular senescence is a critical program to elicit sustained tumor regression upon MYC inactivation. The potential importance of cellular senescence programs in murine and human T-ALL has been described [7, 40]. It was surprising that p53 was less important than p19ARF in regulating cellular senescence. Moreover, p19ARF contributed to senescence induction following MYC inactivation in a p53-independent manner. Previously, we have shown shutdown of angiogenesis is also important for sustained regression of tumors [8]. Our results further confirm p53 loss impeded the ability of MYC inactivation to shutdown angiogenesis. We now show p19ARF loss, but perhaps to a lesser extent, also impeded the ability of MYC inactivation to shutdown angiogenesis.

We observed that p19ARF and p53 loss differentially altered transcriptome of lymphoma cells, as examined in microarray analysis (Supplementary Figure 2A and 2B). Pathway analysis revealed specifically some senescence pathway genes fail to be induced in p19ARF−/− tumors, but not in MYC or MYC p53−/− tumors (Figure 6C). Similarly, a recent study observed oncogenic RAS induces senescence through p19ARF-dependent, but p53- and p16INK4A-independent, transcriptional changes in mouse embryonic fibroblasts [41].

We speculate p19ARF, but not p53, facilitates transcriptional upregulation of senescence pathway genes. p19ARF commands a plethora of p53-independent functions. Extensive physical and functional interactions of p19ARF with various transcription factors, such as E2F1, NFκB, HIF1α, and AR, and its ability to retard ribosome biogenesis through NPM1, NCL, and DDX5, may facilitate p19ARF's influence on transcriptome and its contribution to MYC inactivation induced senescence in a p53-independent manner [28, 42–44]. In contrast, prior reports describe that p19ARF and p53 both regulate cellular senescence [18, 19, 45]. We note that in a melanoma model p19ARF, but not p53, is the most important determinant of premature senescence [46]. We infer that p19ARF is similarly the determinant regulator of cellular senescence in T-ALL.

Our pathway analysis specifically implicated macrophage activation and infiltration associated pathways as being upregulated in WT or p53−/−, but not p19ARF−/−, upon MYC inactivation. In turn, we showed by immunohistochemical staining that p19ARF−/− tumors fail to recruit macrophages at the tumor site. Our results suggest that p19ARF plays a critical role in the regulation of gene products required to elicit macrophage recruitment to the tumor site upon oncogene inactivation. We surmise these innate immune effectors are likely to play a role in remodeling of tumor microenvironment. In tumors transplanted in syngeneic hosts, p19ARF loss in the tumor cells only was sufficient to block macrophage infiltration. We believe that ARF loss in the tumor, not in the host, is responsible for the decrease in macrophage infiltration. However, in mice with primary tumors we cannot rule out that p19ARF status may have affected macrophages as well. Recently, p19ARF loss in macrophages has been linked to deficiency in the induction of cytokines and chemokines and impairment of inflammatory responses [36]. Analysis of microarray data revealed that p19ARF loss resulted in many changes in expression of genes associated with the innate immune programs (Figure 6E). In turn, this was associated with the inability of MYC inactivation to recruit macrophages. It is likely that these broad changes in innate immune programs, rather than any expression change in a single gene, is responsible for the influence of p19ARF on macrophage infiltration. Our finding is especially significant because it connects the loss of p19ARF to an incomplete immune response in regressing tumors.

Our data illustrate that p19ARF regulates self-renewal and senescence programs that may be generally relevant to the therapeutic response of cancers. Many prior results suggest the importance of p19ARF to the regulation of cellular senescence programs [18–21, 47]. One earlier report claimed p19ARF not to be important to senescence induced by cytotoxic chemotherapy [48]. One explanation for this difference is the mechanism of cellular senescence induced by cytotoxic chemotherapy may be different from the inactivation of an oncogene. Alternatively, differences in the types of tumor models may account for these results. The loss of p19ARF correlates with clinical outcome in human patients with ALL. Frequent loss of p19ARF locus at relapse in ALL patients has been previously documented [49]. The p19ARF associated gene signature we identified predicts disease-free survival in ALL patients. Notably, the loss of p19ARF in a breast cancer model leads to relapse following targeted therapy [12]. Experimentally, loss of p19ARF or p53 has been shown to block the ability of Gleevec to inhibit Bcr-Abl induced tumors [13]. Our results suggest that p19ARF loss may impede a therapeutic response by blocking the ability of oncogene inactivation to elicit cellular senescence and an innate immune response.

Cellular senescence programs are critical general features in the therapeutic response of cancers [50]. Similarly, the innate immune response is an important part of the therapeutic response. Our observations elaborate the potential importance of p19ARF as an essential regulator of cellular senescence programs as well as an innate immune response elicited upon MYC oncogene inactivation in a primary transgenic model.

## METHODS

### Mouse experiments

Eμ-tTA (83), TRE-MYC (36), p19ARF knockout (St. Jude Children's Research Hospital, TN, USA), p53 knockout (Baylor College, Texas, USA) mouse lines were used. In p19ARF knockout mouse, only exon 1β is ablated, leaving p16INKA (exons 1α, 2 and 3) and tandemly linked p15INK4B gene intact [51]. p19ARF knockout mice were previously shown, by PCR and IP/WB, to express wild-type p16INK4A. Through crosses, we generated MYC (Eμ-tTA TRE-MYC), MYC p19ARF−/− (Eμ-tTA TRE-MYC p19ARF−/−) and MYC p53−/− mice (Eμ-tTA TRE-MYC p53−/−). All animal experiments were performed following the guidelines from Administrative Panel on Laboratory Animal Care at Stanford University (Protocol 8114, 10563, 14045). All mouse lines were maintained in FVB/N background. To switch off MYC expression, transgenic animals and animals bearing subcutaneous tumors were supplied with 200ug/ml doxycycline in drinking water. *p*-values for survival curves were calculated using (Mantel-Cox) log rank test in Graph Pad Prism 5.

### Cell culture and immunoassays

Tumor-derived cell lines were generated as described previously [3]. To suppress MYC transgene expression, 20ng/ml doxycycline was added to the medium. p19ARF shRNA target sequence (CGCTCTGGCTTTCGTGAACAT) [47] lies in the exon 1β of CDKN2A gene, hence it is specific to p19ARF. shRNA was retrovirally delivered in MSCV-LMP vector. Cells expressing shRNA were selected using GFP FACS and puromycin.

Western blotting and immunofluorescence were performed as described [7]. Pictures were taken with 40x objective on Nikon E800 and Nikon E1000M microscopes using Spot Advanced software. For flow cytometry, cells were washed in PBS, incubated in FITC- or PE-conjugated antibody cocktail (1:200) and propidium iodide (10 μg/ml), analyzed in FACScan with CellQuest and FlowJo software.

Senescence associated beta-galactosidase, Ki67 and TUNEL assays were performed as previously described [6]. Multiple tumors per genotype were stained and analyzed in every experiment. Images were quantified using MetaMorph image analysis software. *p*-values were calculated using a two-tailed, unpaired *t*-test.

### Microarray and data analysis

RNA was isolated from tumor-derived cells cultured *in vitro* where MYC is on or MYC had been turned off for 24 hours. Illumina WG-6 murine arrays were read using Illumina Bead Studio 3.4. The array data were filtered in Genespring GX 10. “MYC on” over “MYC off” expression level ratios were calculated for each gene (two-tailed, paired, t test *p* = 0.05, ≥ 2-fold change, no multiple testing correction): MYC on/MYC off (*n* = 6), MYC on p19−/−/MYC off p19−/− (*n* = 3), and MYC on p53−/−/MYC off p53−/− (*n* = 2). These gene lists were then compared to ascertain significant genes that were unique to each tumor type and that overlapped between various sample combinations. This created 7 independent gene expression signatures: MYC, MYC p19−/−, MYC p53−/−, MYC ∩ MYC p19−/−, MYC ∩ MYC p53−/−, MYC p19−/− ∩ MYC p53−/−, and MYC ∩ MYC p19−/− ∩ MYC p53−/−.

Assessment of the MYC target gene expression levels in each group was done using a list of MYC target genes from the MYC Target Gene Database on http://www.myc-cancer-gene.org.

Data for two ALL data sets (GSE18497 and GSE11877) were used to create a ranked list of z-scores, and a pre-rank GSEA algorithm was employed to test whether each of the gene signatures derived from our murine ALL models was enriched for poor prognosis (positive z-score) or good prognosis (negative z-score) genes.

## SUPPLEMENTARY METHODS


