# Supplementary material for: p19ARF is a critical mediator of both cellular senescence and an innate immune response associated with MYC inactivation in mouse model of acute leukemia
Source: Oncotarget. 2015 Jan 13;6(6):3563–77. doi: 10.18632/oncotarget.2969 (PMC4414137; doi:10.18632/oncotarget.2969)
Supplement: Supplementary file 1 [file oncotarget-06-3563-s001.pdf]

SUPPLEMENTARY METHODS

Cell culture

Cells were extracted from thymus of lymphoma-bearing mice and maintained in RPMI supplemented with 20% FBS, 1% antibiotic/antimycotic and 50 µM 2-mercaptoethanol. To suppress MYC transgene expression *in vitro* 20 ng/ml doxycycline was added to the growth medium.

Retrovirus constructs and infection

p19ARF shRNA target sequence (CGCTCTGG CTTTCGTGAACAT) lies in the exon 1β of CDKN2A gene, hence it is specific to p19ARF. Target sequence for shRNA was cloned into MSCV-LMP vector (Open Biosystems). Retrovirus was produced using ecotrophic Phoenix cell line (Gary Nolan laboratory, Stanford) and target cells were infected with retrovirus containing medium supplemented with 4 µg/ml hexadimetrine bromide (polybrene). GFP expressing cells were sorted using FACS Vantage. Puromycin resistant cells were selected in medium containing 10 ug/ml puromycin.

List of antibodies

The following antibodies were used for Western blotting, immunohistochemistry and immunofluorescence: Human MYC 9E10 (sc40-X), p19ARF 5C3 (ab26696), p53 (Cell Signaling 2524), p16Ink4a F12 (sc1661), CD31 (ab28364), H3K4 trimethylation (ab8580), and H3K9 trimethylation (ab8898).

The following antibodies were used in flow cytometry: CD3-PE (Pharmingen 01085B), Thy1.1-FITC (BD 554894), CD5-PE (BD 553022), CD4-FITC (BD 553651), CD8α-PE (BD 553033), B220-PE (BD 553090), IgM-FITC (BD 553437), CD19-FITC (Pharmingen 09654D), Gr1-FITC (BD 553127), and Mac1α-PE (BD 553311). For apoptosis, AnnexinV-PE (BD 556422).

Senescence associated beta-galactosidase (SA-β-Gal) assay

Cells and OCT-frozen tissue sections were fixed in 0.5% glutaraldehyde in PBS, washed in PBS/1 mM $MgCl_2$, and stained at 37°C with 0.1% X-gal (bromo-chloro-indolyl-galactopyranoside) in PBS/1 mM $MgCl_2$/1 mM $K_4Fe(CN)_6$/1 mM $K_3Fe(CN)_6$.

Microarray and data analysis

RNA was isolated from tumor-derived cell lines where MYC is on or MYC had been turned off for 24 hours. RNA was run on Illumina WG-6 murine arrays. The arrays were read using Illumina Bead Studio 3.4. The array data were filtered in Genespring GX 10. "MYC on" over "MYC off" expression level ratios were calculated for each gene (two-tailed, paired, t test $p = 0.05$, ≥ 2-fold change, no multiple testing correction): MYC on/MYC off ($n = 6$), MYC on p19−/−/MYC off p19−/− ($n = 3$), and MYC on p53−/−/MYC off p53−/− ($n = 2$). These gene lists were then compared to ascertain significant genes that were unique to each tumor type and that overlapped between various sample combinations. This created 7 independent gene expression signatures: MYC, MYC p19−/−, MYC p53−/−, MYC ∩ MYC p19−/−, MYC ∩ MYC p53−/−, MYC p19−/− ∩ MYC p53−/−, and MYC ∩ MYC p19−/− ∩ MYC p53−/−.

Assessment of the MYC target gene expression levels in each group was done using a list of MYC target genes from the MYC Target Gene Database on http://www.myc-cancer-gene.org. MYC target gene expression data was clustered via hierarchal clustering for both genes and samples. MYC target genes were split into transactivated and transrepressed categories based on information on http://www.myc-cancer-gene.org.

Assessment of the macrophage associated gene differences between the groups was done using the ToppGene algorithm on https://toppgene.ccgmc.org.

Survival analysis of all data based on murine gene signatures

Raw expression data for two ALL data sets (GSE18497 and GSE11877) from Gene Expression Omnibus (GEO) were converted to log (2), imputed for missing values, and quantile normalized. z-score for each microarray probe was calculated by univariate Cox regression between the expression level of the probe and the clinical outcomes (relapse free survival (RFS), overall survival (OS) and event free survival (EFS)). Finally, z-scores were averaged for multiple probes to yield a single survival z-score for each gene.

A ranked list of z-scores was created, and a pre-rank GSEA algorithm was employed to test whether each of the gene signatures derived from our murine ALL models was enriched for poor prognosis (positive z-score) or good prognosis (negative z-score) genes, including up- and downregulated genes from: MYC, MYC p19−/−, MYC p53−/−, MYC ∩ MYC p19−/−, MYC ∩ MYC p53−/−, MYC p19−/− ∩ MYC p53−/−, and MYC ∩ MYC p19−/− ∩ MYC p53−/−. Only the genes that are uniquely upregulated in MYC p19ARF−/− samples showed significant correlation to poor prognosis in the z-score analysis. We used MYC p19ARF−/− gene signature to stratify patients into high- and low-risk groups in two

publically available human ALL cohorts (the Willman laboratory's Children's Oncology Group Study 9906 for High-Risk Pediatric ALL (GSE11877) and the De Ridder laboratory's Diagnosis-Relapse in ALL (GSE18497)). The upregulated MYC p19ARF−/− gene signature was used to perform a k-means clustering analysis to group samples into two: patients who have high expression of signature genes) and patients who do not have high expression of the genes. Kaplan Meier curves for OS and EFS were plotted for these two groups the difference between the two stratification groups was statistically assessed by Mantel-Cox log-rank test in Graph Pad Prism 5.

SUPPLEMENTARY FIGURES

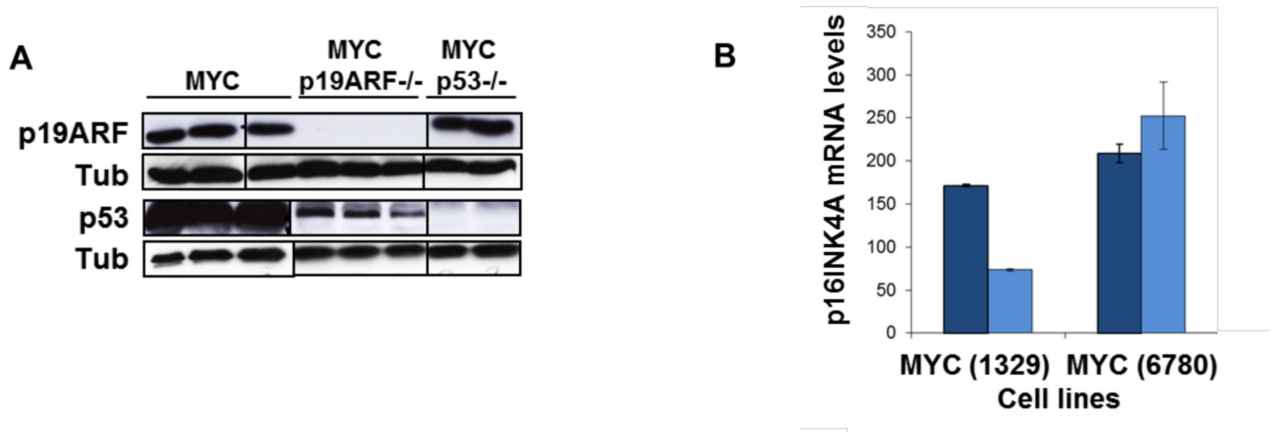

Supplementary Figure 1: p19ARF and p53 status of MYC, MYC p19ARF−/− and MYC p53−/− tumors and p16INK4A mRNA levels. (A) WB showing reduced p53 expression in p19ARF−/− tumors and retention of p19ARF expression in p53−/− tumors. Loading control is alpha tubulin. **(B)** qPCR showing p16INK4A levels after 24 hours of MYC inactivation in MYC induced lymphomas.

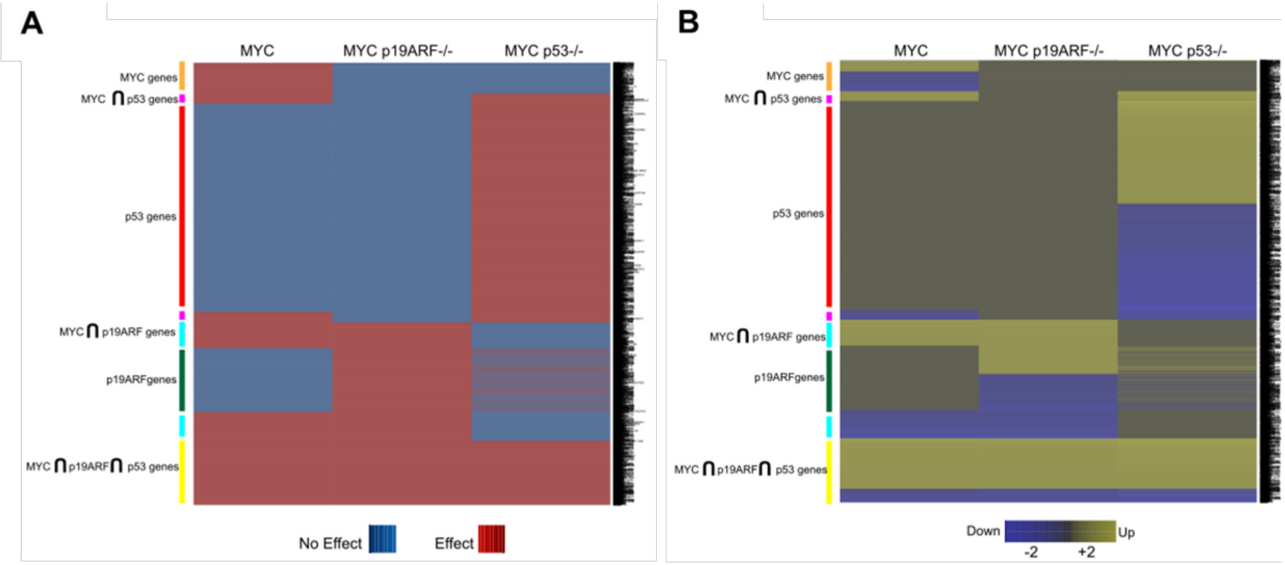

C

Gene Signatures	MYC	MYC/p19ARF	MYC/p53	% of genes	%_MYCgenes_UP	%_MYCgenes_DOWN
MYC	1	0	0	6.9	2.4	4.5
MYC/p53	1	0	1	4.6	2.2	2.4
p53	0	0	1	47.2	0	0
MYC/p19ARF	1	1	0	12.3	5.8	6.5
p19ARF	0	1	0	9.6	0	0
p19ARF/p53	1	0	1	4.8	0	0
MYC/p19ARF/p53	1	1	1	14.5	11.4	3.1

1 = Differentially expressed , 0 = Not differentially expressed

Supplementary Figure 2: Microarray analysis of all significant transcriptional changes upon MYC inactivation reveals influence of p19Arf and p53 status on transcriptome. (A) Heatmap summary of sorted genes that significantly change upon MYC inactivation in MYC, MYC p19ARF−/−, and MYC p53−/−. A gene is coded red as differentially expressed (effect) compared to the control under a given tumor system or blue if not differentially expressed (no effect). (Left) The gene list names refer to the gene signatures MYC, MYC ∩ MYC p53−/−, MYC p53−/−, MYC ∩ MYC p19−/−, MYC p19−/−, and MYC ∩ MYC p19−/− ∩ MYC p53−/− color coded as orange, pink, red, blue, green and yellow respectively. Each row within each signature corresponds to the genes belonging to that signature **(B)** A corresponding heatmap of the same genes in (A) showing both transactivation and transrepression effects. All genes coded in blue are more than or equal to 2 fold change down regulated in the corresponding tumor model and genes coded in yellow are more than or equal to 2 fold change up regulated. **(C)** A corresponding table of percentage of genes in addition to the percentage of MYC transactivation and transrepression genes for all 7 gene signatures in C.

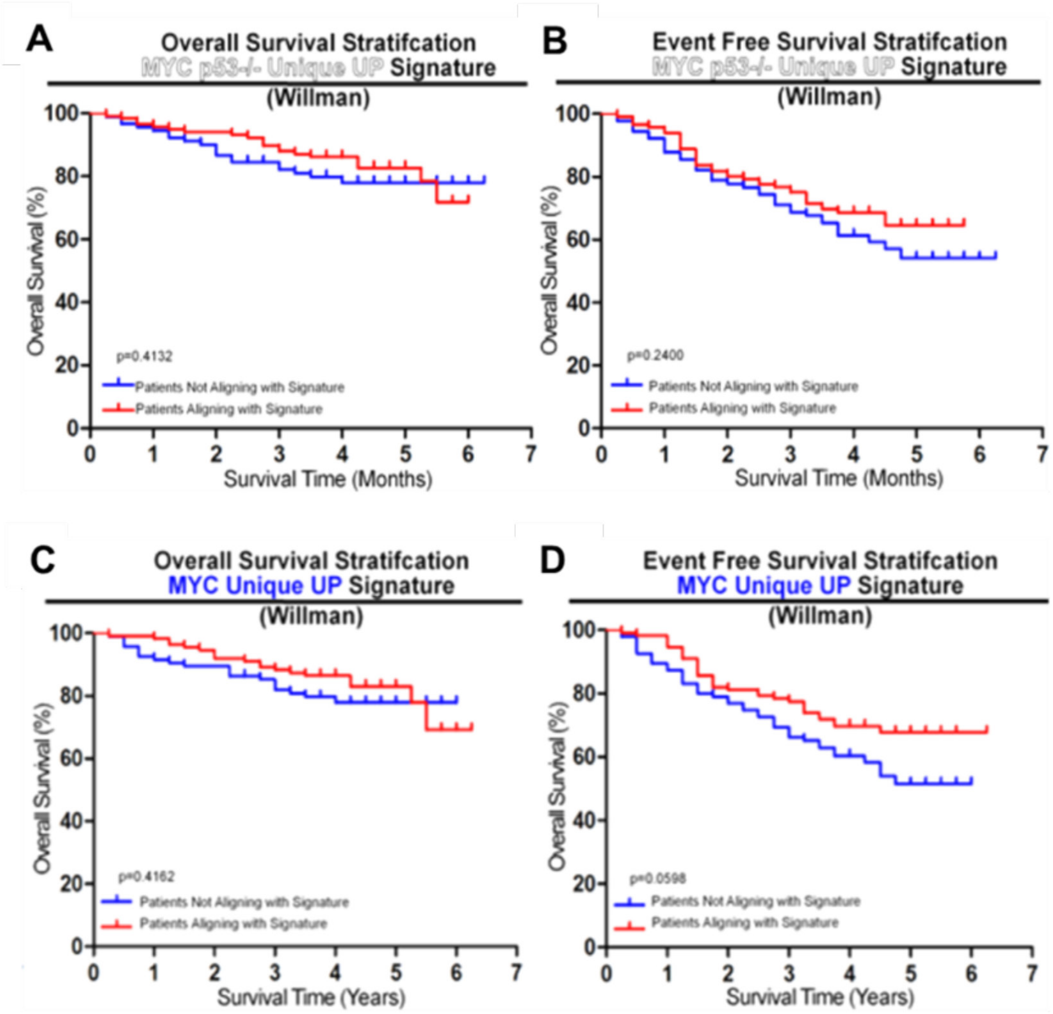

Supplementary Figure 3: MYC and MYC p53−/− gene signatures fail to stratify patients for overall survival or event-free survival. **(A, B)** Human B-ALL patients from the cohort collected by the Willman laboratory (GSE11877) for the Children's Oncology Group Study 9906 for High-Risk Pediatric ALL were divided into two groups by k-means clustering utilizing the MYC p53−/− unique gene signature and then stratified via Kaplan-Meier survival analysis for overall survival and event free survival, revealing that there was no significant difference between the two patient groups based on overall survival or event free survival. **(C, D)** Human B-ALL patients from the cohort collected by the Willman laboratory (GSE11877) for the Children's Oncology Group Study 9906 for High-Risk Pediatric ALL were divided into two groups by k-means clustering utilizing the MYC unique gene signature and then stratified via Kaplan-Meier survival analysis for overall survival and event free survival, revealing that there was no significant difference between the two patient groups based on overall survival or event free survival.
